# Changes in Cigarette Smoking Prevalence After Passing a Recreational Cannabis Legalization Law Without Retail Sales: Evidence From Virginia, U.S.

**DOI:** 10.1016/j.focus.2026.100482

**Published:** 2026-01-20

**Authors:** Samuel Asare, Johann Lee Westmaas, Nigar Nargis

**Affiliations:** Tobacco Control Research, Department of Surveillance, Prevention, & Health Services Research, American Cancer Society, Atlanta, Georgia

**Keywords:** Recreational cannabis law, cannabis possession laws, cannabis retail dispensary sales, cigarette smoking

## Abstract

**Introduction:**

Several U.S. states have passed recreational cannabis legalization laws that permit adult use, possession, cultivation, sharing, and retail (dispensary) sales of cannabis. Considering the variability in cannabis laws across states and that cannabis use is associated with cigarette smoking, state-specific analyses of the impact of recreational cannabis legalization laws on cigarette smoking are needed. The authors examined changes in cigarette smoking prevalence in Virginia after the passage of a recreational cannabis legalization law in April 2021 that permitted adult use, possession, sharing, and cultivation of cannabis but not retail dispensary sales.

**Methods:**

This cross-sectional study utilized data from 992,615 participants aged ≥18 years from the Behavioral Risk Factor Surveillance System survey conducted annually from 2015 to 2023. The authors used a difference-in-differences method to compare cigarette smoking prevalence in Virginia before (January 1, 2015–April 28, 2021) and after (May 1, 2021–December 31, 2023) the passage of its recreational cannabis legalization law with that in 15 states that did not pass any recreational cannabis legalization law as of December 2023. The analysis accounted for sociodemographic characteristics, state-specific time-varying and time-invariant factors, seasonality, and confounders that are common across states but change over time.

**Results:**

After the passage of recreational cannabis legalization law, the probability of smoking cigarettes in Virginia, in relation to that in comparison states, increased by 1.4 percentage points (95% CI=0.27, 2.60; *p*=0.02). This estimate was equivalent to a 9.5% relative increase in smoking prevalence over the prerecreational cannabis legalization law level of 15.1%. Stratified analyses by specific sociodemographic groups indicated that, in Virginia compared with comparison states, smoking prevalence increased by 10.2% among males, 64.3% among Hispanics, 19.9% among persons whose education is high school level or lower, and 14.7% among people aged 21–49 years. In contrast, a 61.6% decrease was observed among young adults aged 18–21 years who were not eligible to use recreational cannabis under Virginia’s recreational cannabis legalization law.

**Conclusion:**

Stronger tobacco control measures and integration with cannabis regulations may be needed in Virginia to mitigate increases in cigarette smoking prevalence associated with passage of the recreational cannabis legalization law.

## HIGHLIGHTS

• In 2021, Virginia legalized recreational cannabis use but not retail sales.
• Difference-in-differences analyses indicated that the law increased cigarette smoking.
• Larger increases in cigarette smoking were observed for Hispanic persons.
• Tobacco policies may mitigate cigarette smoking associated with cannabis legalizations.

## INTRODUCTION

Cannabis legalization for recreational purposes has been increasing globally (e.g., Australia, Canada, South Africa, Thailand, U.S.).^1^^,^^2^ Although in the U.S., cannabis is still an illicit substance at the federal level, since 2012, several states have passed recreational cannabis laws (RCLs) to permit adult (aged ≥21 years) use of cannabis for recreational purposes. This has coincided with a dramatic increase in cannabis use in the U.S. Between 2002 and 2014, past-month cannabis use among persons aged ≥12 years had increased slowly from 6.2% to 8.4%^3^; however, by 2023, past-month use of cannabis reached 15.4%.^4^

In most U.S. states, RCLs cover decriminalization of cannabis possession, sharing of cannabis, cultivation of cannabis plants, and allowance of retail (dispensary) sales. However, the legalization of retail sales of cannabis can occur either concurrently with the passage of an RCL or at a later date. In April 2021, Virginia became the first U.S. Southern state to pass an RCL.^5^ The state permitted adults aged ≥21 years to possess or share up to 1 ounce of cannabis.^6^ It also permitted the cultivation of up to 4 cannabis plants in the owner’s primary residence for personal use. Importantly, retail dispensary sales of cannabis were not included in the law’s passage in 2021; that was scheduled for January 1, 2024 but deferred to May 1, 2026.^7^ To ensure compliance with the RCL regulations in Virginia, misdemeanor or felony offenses, punishable by fines and/or imprisonment, are applied on the basis of the severity of the violations.^6^

Passage of RCLs is generally associated with increases in cannabis use,^8^^,^^9^ and the effects of Virginia’s RCL on cannabis use may be no different on the basis of the authors’ exploratory examination of the National Survey on Drug Use and Health data from 2012 to 2023. Excluding pandemic years (because those data are unavailable), cannabis use in Virginia in the past 30 days among persons aged ≥12 years varied between 6.9% in 2014–2015^10^ and 7.9% in 2018–2019^11^ but increased to 13.3% from 2022–2023^12^ after RCL was passed. Because RCLs increase the acceptability of a formerly illegal substance, they may lead to changes in demand, use, and prices of other related substances if these were used as complements or substitutes for cannabis.^13^^,^^14^ This may be especially the case for cigarette smoking, given the positive associations between cannabis and tobacco use.^15^^,^^16^ Previous studies have found that cannabis use is associated with increased cigarette smoking initiation, decreased smoking cessation, and increased smoking relapse among adults.^15^^,^^17^, ^18^, ^19^

However, there is no consensus on whether RCLs influence cigarette smoking.^8^^,^^20^, ^21^, ^22^, ^23^, ^24^ Previous studies found that RCLs either reduced tobacco use,^20^^,^^21^ were not associated with adult cigarette smoking,^8^^,^^22^^,^^23^ or were associated with increased cigarette use.^24^ However, most of these previous investigations focused on early-adopting RCL states, and only 1 study examined the effects of both early and recent RCLs.^22^ Moreover, these investigations have treated RCLs, with or without all the components, as similar or uniform policies across states.^8^^,^^20^, ^21^, ^22^, ^23^, ^24^ In practice, RCLs differ in the quantity of cannabis permissible to possess or share, penalties for violations, the number of plants allowed to be cultivated at home, amendments of the laws over time, and monitoring and enforcement of the laws. This suggests that the inconsistencies in previous study findings may be partly due to differences across states in the breadth of the law and/or the sequence of implementation of certain parts of the law. Thus, an investigation that considers each RCL in every state as unique and different from those in other states is warranted.

Permitting retail sales of cannabis is an important component of an RCL because it is widely assumed that the increased accessibility or availability of cannabis through retail dispensaries contributes significantly to increases in cannabis use.^8^^,^^9^ To the extent that cannabis and cigarettes are complements or substitutes, RCLs that include concurrent retail dispensary sales would be expected to influence both cannabis use and cigarette smoking. However, an RCL that is yet to permit retail dispensary sales and allow easy access may experience relatively smaller or minimal increases in cannabis use that, in turn, would be reflected in cigarette smoking prevalence. Although some states have added retail dispensary provisions to their RCLs concurrently or over time, Virginia is unique in passing an RCL in 2021 that still lacks such a component. This presents a rare quasiexperimental setup to assess whether the passage of a recent RCL without allowing retail sales of cannabis would lead to changes in cigarette smoking prevalence. Therefore, the authors examined changes in cigarette smoking prevalence in Virginia that passed RCL without retail dispensary sales of cannabis compared with changes in prevalence in 15 states that did not pass or implement any RCL in the time periods before and after Virginia passed its RCL in 2021. The authors were also interested in determining whether the effects of the law differed for population subgroups defined by sociodemographic characteristics (i.e., sex, race/ethnicity, education, and age).

## METHODS

### Study Sample

The data were from the nationally representative, cross-sectional Behavioral Risk Factor Surveillance System (BRFSS) survey of participants aged ≥18 years, conducted annually by the U.S. Centers for Disease Control and Prevention from 2015 to 2023.^25^

### Measures

For the outcome, the survey asked, *Have you smoked at least 100 cigarettes in your entire life? (Yes / No). Do you now smoke cigarettes every day, some days, or not at all? (Every day, Some days, Not at all, Don’t know / Not sure, Refused).* The authors defined current cigarette smoking as a binary outcome, with a value of 1 assigned if the individual had smoked ≥100 cigarettes and smoked every day or some days and 0 otherwise.

In terms of covariates, the survey also collected data on the sociodemographic characteristics of respondents, for which the authors controlled for in the analyses. These included age (in years) and indicator variables for sex (male / female), marital status (single versus married and cohabitating), race/ethnicity (African American, Hispanic, White, and other), educational level (no high-school diploma, high-school diploma, some college, and college degree or higher), and household income (<$10,000; $10,000–$14,999; $15,000–$19,999; $20,000–$24,999; $25,000–$34,999; $35,000–$49,999; $50,000–$74,999; ≥$75,000).^22^ Indicator variables were also created for respondents with missing information on marital status, race/ethnicity, education, and household income.

State-level time-varying covariates from supplementary data sources included tobacco control and related policies, state welfare programs and sociopolitical environment, macroeconomic environment, cannabis-related policies, and coronavirus disease 2019 (COVID-19) pandemic covariate that are correlated with cigarette smoking or cannabis regulations.^8^^,^^26^^,^^27^ The tobacco control and related policies were inflation-adjusted per-pack excise tax on cigarettes,^28^ presence of E-cigarette taxes,^29^ and inflation-adjusted beer tax per gallon.^30^ The remaining variables were for the state welfare programs and sociopolitical environment (i.e., Affordable Care Act Medicaid expansion^31^ and presence of a Democrat governor^32^^,^^33^), the state macroeconomic environment (as reflected in inflation-adjusted minimum wage per hour^34^ and unemployment rate^35^), presence of a medical cannabis legalization law,^36^ and the presence of legalization of retail sales of cannabis in the neighboring states to account for spillover effects.

### Statistical Analysis

The authors analyzed the full sample and performed stratified analyses by some subpopulation groups (sex, race/ethnicity, education, and age). The study did not require IRB/ethics review because the BRFSS data were publicly available and deidentified. The authors followed STROBE reporting guidelines and reported marginal effects from probit estimations for interpretation. All observations were weighted using sampling survey weights to make the samples representative of state populations, and SEs were clustered at the state level. The authors used Stata, Version 18.0 (StataCorp LLC), to conduct analyses.

A difference-in-differences specification was used to compare cigarette smoking prevalence in Virginia from before (January 1, 2015–April 28, 2021) and after (May 1, 2021–December 31, 2023) the passage of its RCL with smoking prevalence in 15 comparison states that did not pass any RCLs as of December 2023. The authors estimated the following difference-in-differences specification (Equation 1):

$$Y_{ismt}=\alpha_{0}+\alpha_{1}RCL_{smt}+\alpha_{2}MCL_{smt}+\alpha_{3}Spillover_{smt}+\beta X_{ismt}+\varphi Z_{smt}+\;\gamma_{s}+\gamma_{m}+\gamma_{t}+\xi_{ismt},$$

where $Y_{ismt}$ is current cigarette smoking (=1 if individual *i* in state *s* smoked cigarettes in month *m* of year *t* and 0 otherwise), and $RCL_{smt}$ is a dummy for recreational cannabis legalization law in state *s* in month *m* in year *t* (=1 if the participant was interviewed in Virginia from May 1, 2021 to December 31, 2023 and 0 otherwise). $MCL_{smt}$ is dummy variable for medical cannabis legalization law. $Spillover_{smt}$ is dummy variable for the presence of legalization of retail sales of cannabis in the neighboring state. $X_{ismt}$ is a vector of sociodemographic characteristics (sex, race/ethnicity, education, age, marital status, and household income). $\gamma_{s}$ is a vector of state-fixed effects. $\gamma_{m}$ is vector of survey-month fixed effects. $\gamma_{t}$ is a vector of year-fixed effects. $Z_{smt}$ is a vector of state-level time-varying characteristic.

The coefficient $\alpha_{1}$ in Equation 1 represents the difference in adjusted cigarette smoking prevalence between Virginia and comparison states from before to after Virginia passed its RCL under the assumption of parallel trends. The comparison group comprised 15 states in the Southern and Northeast regions of the U.S. Census Division that did not pass any RCLs before and during the sample period.^37^ They were Alabama, Arkansas, Florida, Georgia, Kentucky, Louisiana, Mississippi, New Hampshire, North Carolina, Oklahoma, Pennsylvania, South Carolina, Tennessee, Texas, and West Virginia.

The parallel trends assumption requires no differences in the evolution of cigarette smoking prevalence between Virginia and comparison states in the post-RCL period in the absence of an RCL. Because this cannot be tested, the authors used an event study to determine whether cigarette smoking prevalence patterns between Virginia and comparison states were similar before Virginia passed its RCL. The authors estimated the specification below (Equation 2):

$$Y_{ismt}=\;\tau_{0}+\tau_{2015}\;VA\times Year_{2015}+\;\tau_{2016}\;VA\times Year_{2016}+\ldots +\;\tau_{2019}\;VA\times Year_{2019}+\tau_{2021}\;VA\times Year_{2021}+\tau_{2022}\;VA\times Year_{2022}+\tau_{2023}\;VA\times Year_{2023}+\tau_{1}MCL_{smt}+\tau_{2}Spillover_{smt}+\theta X_{ismt}+\delta Z_{smt}+\lambda_{s}+\gamma_{m}+\;\lambda_{t}+\mu_{ismt},$$

where VA is a dummy for residents in Virginia (=1 if the respondent was surveyed in Virginia and 0 otherwise), and $Year_{J}$ (for *J*=2015, 2016, …, 2023) is year dummy variables.

The coefficients $\tau_{2015}$, $\tau_{2016}$, …, $\tau_{2023}$ were of interest and represented differences in adjusted smoking prevalence between Virginia and comparison states relative to the baseline (2020) level of no difference. The assumption of parallel trends was descriptively satisfied in these data if all the ${\hat{\tau }}_{J}$s (for *J*=2015, 2016, …, 2019) were not statistically significant.

The authors conducted 3 sets of sensitivity analyses to test the robustness of their results. Virginia passed its RCL in 2021, approximately 12 months after the onset of the COVID-19 pandemic in the U.S. The first set tested for COVID-19 pandemic–related influences on the estimates by excluding data in 2020; restricting data to the period after the onset of the pandemic; or including a covariate variable for state-level cumulative COVID-19 infection rate per 1,000 persons. In the second set, the authors demonstrated that the results are robust to the choice of comparison states by restricting the comparison states to those of the Southern region of the U.S. Census Division; including all 27 U.S. states that did not pass RCLs; or excluding data from all the neighboring states of Virginia from the analysis. In the third set, the authors excluded data from 2021; allowed for an implementation lag so that the post-RCL period began in January 2022; or allowed for possible changes in prevalence in anticipation of the RCL by using a post-RCL period that began in January 2021.

## RESULTS

Of the 992,615 individuals included in the analysis, 80,270 were from Virginia (55,502 from before and 24,768 from after the passage of RCL), whereas 912,345 were from the 15 comparison states (675,771 from before and 236,574 from after the enactment of RCL). The mean age of respondents was 47.82 (SD=18.16) years. Approximately 48.46% of individuals in the sample were males, and 51.54% were females. Significant differences were observed between Virginia and comparison states for some sociodemographic characteristics (e.g., persons identified as other race/ethnicity, educational levels, household income groups) and state-level covariates (e.g., cigarette taxes, minimum wage, presence of Medicaid expansion) (Table 1).

**Table 1:** Sociodemographic Characteristics Among Adults Aged ≥18 Years in Virginia and Comparison States From 2015 to 2023^a^

Characteristics	Mean (SE)		Mean difference (SE)	*p*-value
	Virginia	Comparison states^b^		
Current smoking	14.02 (0.00)	16.45 (0.88)	−2.42 (−4.29, −0.56)	0.01
Individual characteristics				
Age, years	47.35 (0.0)	47.86 (0.63)	−0.50 (0.63)	0.4
Sex, %				
Male	48.69 (0.00)	48.43 (0.21)	0.26 (0.21)	0.2
Female	51.31 (0.00)	51.57 (0.21)	−0.26 (0.21)	0.2
Marital status, %				
Single	44.23 (0.00)	45.39 (0.58)	−1.16 (0.58)	0.06
Married or cohabitating	55.77 (0.00)	54.61 (0.58)	1.16 (0.58)	0.06
Missing data^c^	0.79 (0.00)	0.74 (0.07)	0.05 (0.07)	0.5
Race and ethnicity, %				
African American	17.94 (0.00)	16.03 (2.10)	1.91 (2.10)	0.37
Hispanic	8.76 (0.00)	15.98 (5.34)	−7.22 (5.36)	0.2
White	61.85 (0.00)	60.24 (4.81)	1.61 (4.81)	0.7
Other^d^	9.42 (0.00)	5.76 (0.53)	3.66 (0.53)	<0.001
Missing data^c^	2.03 (0.00)	1.99 (0.11)	0.04 (0.11)	0.7
Educational level, %				
No high-school diploma	10.63 (0.00)	13.79 (0.82)	−3.16 (0.82)	0.002
High-school diploma	25.16 (0.00)	29.58 (1.15)	−4.42 (1.15)	0.002
Some college	29.20 (0.00)	30.45 (0.51)	−1.25 (0.51)	0.03
College degree or higher	34.66 (0.00)	25.78 (0.56)	8.88 (0.56)	<0.001
Missing data^c^	0.34 (0.00)	0.39 (0.03)	−0.05 (0.03)	0.2
Household income, %				
<$10,000	2.75 (0.00)	4.20 (0.19)	−1.45 (0.19)	<0.001
$10,000–$14,999	2.61 (0.00)	3.79 (0.13)	−1.18 (0.13)	<0.001
$15,000–$19,999	4.23 (0.00)	5.94 (0.16)	−1.70 (0.16)	<0.001
$20,000–$24,999	5.93 (0.00)	7.42 (0.20)	−1.49 (0.20)	<0.001
$25,000–$34,999	7.92 (0.00)	9.48 (0.14)	−1.55 (0.14)	<0.001
$35,000–$49,999	9.99 (0.00)	11.26 (0.23)	−1.26 (0.23)	<0.001
$50,000–$74,999	12.66 (0.00)	12.66 (0.26)	−0.00 (0.26)	1
≥$75,000	37.06 (0.00)	27.08 (0.91)	9.98 (0.91)	<0.001
Missing data^c^	16.84 (0.00)	18.19 (0.51)	−1.35 (0.51)	0.02
State-level characteristics				
Per pack tax on cigarettes, in February 2020, $	0.44 (0.00)	1.24 (0.20)	−0.80 (0.20)	0.001
Beer taxes, in February 2020, $	0.27 (0.00)	0.51 (0.12)	−0.24 (0.12)	0.06
Minimum wage, in February, 2020 $	8.92 (0.00)	7.92 (0.33)	0.90 (0.33)	0.02
Unemployment rate, %	3.65 (0.00)	4.62 (0.11)	−0.97 (0.11)	<0.001
Presence of E-cigarette taxes, %	39.61 (0.00)	26.38 (12.24)	13.24 (12.24)	0.3
Presence of Medicaid expansion, %	55.52 (0.00)	23.95 (11.86)	31.58 (11.86)	0.02
Democrat governor, %	75.70 (0.00)	23.22 (12.39)	52.48 (12.39)	0.001
Medical cannabis legalization laws, %	35.22 (0.00)	54.87 (14.01)	−19.66 (14.01)	0.2
Presence of legalization of retail sales of cannabis in the neighboring state, %	0.00 (0.00)	11.17 (4.65)	−11.17(4.65)	0.03
Observations, *n*	80,270	912,345	NA	NA

a The BRFSS data used to compute the statistics were available at the individual level. The means and differences with their SEs were computed using the *svy* command in STATA, Version 18.0 (StataCorp LLC).

b Comparison states were Alabama, Arkansas, Florida, Georgia, Kentucky, Louisiana, Mississippi, New Hampshire, North Carolina, Oklahoma, Pennsylvania, South Carolina, Tennessee, Texas, and West Virginia.

c Because approximately 16% of respondents did not provide information on household income, the authors categorized it as missing and included it in the regressions as a separate category of household income. The authors also created three other categories to separately identify individuals with missing information on marital status, race/ethnicity, and education.

d Other included the following race/ethnicity identified in the BRFSS survey: American Indian or Alaska Native, Asian, Pacific Islander, and any other race not listed. BRFSS, Behavioral Risk Factor Surveillance System; NA, not applicable.

**Figure 1:**
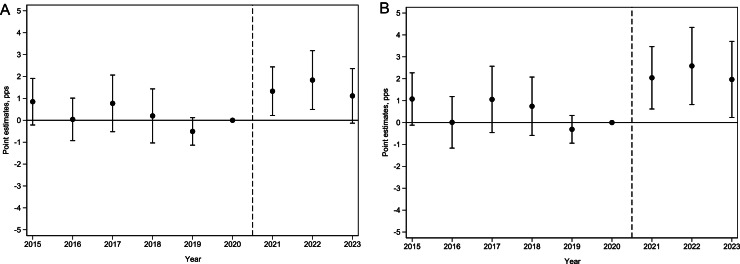
Event study estimates. (A) All individuals (aged ≥18 years). (B) RCL eligible population (aged ≥21 years) *Note*: The point estimates (circles) with 95% CIs (error bars) are the difference-in-differences estimates (in percentage points) on the basis of Equation 2.

The event study analysis to evaluate the parallel trends assumption (Equation 2) indicated no differences in trends for the adjusted prevalence of cigarette smoking between Virginia and comparison states in all years preceding Virginia’s RCL passage in 2021 for adults aged ≥18 years (Figure 1A). This finding was robust after including only adults aged ≥21 years eligible to purchase tobacco products (Figure 1B).

The estimated difference in smoking prevalence, after adjusting for covariates, between Virginia and the comparison states from before to after the passage of its RCL in 2021 was 1.44 percentage points (pps) (95% CI=0.27, 2.60; *p*=0.02). This was equivalent to a 9.5% increase in smoking prevalence in Virginia relative to the state’s pre-RCL smoking prevalence of 15.1% (Table 2). The estimate was slightly higher after excluding young adults aged 18–20 years from the sample (1.74 pp; 95% CI=0.22, 3.33: *p*=0.03). Sensitivity analyses indicated that the difference-in-differences estimate was generally not affected by the COVID-19, selection of comparison states, or adjustments that accounted for an implementation lag or anticipation of the RCL.

**Table 2:** Estimates of the Association Between Recreational Cannabis Legalization Law and Current Cigarette Smoking in Virginia Among Adults Aged ≥18 Years From 2015 to 2023

Sample	Difference-in-differences estimate (95% CI), percentage points^a^	Current cigarette smoking prevalence in Virginia before passing RCL, %	Relative changes, %^b^	*p*-value	BRFSS unweightedsample size, *n*
Full sample analysis					
All individuals aged ≥18 years	1.44 (0.27, 2.60)	15.11	9.50	0.02	992,615
Individuals aged ≥21 years	1.74 (0.22, 3.33)	15.56	11.16	0.03	970,757
Sensitivity to the COVID-19 pandemic					
Inclusion of the COVID-19 infection rate	1.53 (0.28, 2.78)	15.11	10.13	0.02	992,615
Exclusion of data from 2020 due to the COVID-19 pandemic	1.10 (-0.11, 2.30)	15.40	7.13	0.07	893,613
Restricted the sample period to 2020–2023	2.67 (0.87, 4.46)	13.60	19.61	0.004	384,802
Sensitivity to the selection of comparison states					
Only states from the Southern region^c^	1.74 (0.47, 3.00)	15.11	11.49	0.007	891,820
All 27 states without RCL^d^	1.21 (0.24, 2.19)	15.11	8.03	0.01	1,811,158
Excluded data from neighboring states of Virginia^e^	1.23 (0.13, 2.34)	15.11	8.14	0.03	795,485
Sensitivity to the selection of the post-RCL period					
Excluded data from 2021	2.41 (1.22, 3.61)	15.14	15.95	<0.001	900,110
Moved post-RCL period to begin from January 2021	1.51 (0.26, 2.75)	15.11	9.97	0.02	992,615
Moved post-RCL period to begin from January 2022	0.95 (-0.15, 2.01)	14.86	6.41	0.09	992,615

a The difference-in-differences estimates show the association between current cigarette smoking and RCL in Virginia using 15 states from the Northeast and Southern U.S. Census Regions that did not pass RCLs before the end of the sample period in December 2023 as comparison states. Comparison states were Alabama, Arkansas, Florida, Georgia, Kentucky, Louisiana, Mississippi, New Hampshire, North Carolina, Oklahoma, Pennsylvania, South Carolina, Tennessee, Texas, and West Virginia.

b The relative changes in the current smoking prevalence were calculated by dividing the difference-in-differences estimates in the second column by the current smoking prevalence rates in Virginia in the third column and multiplying by 100.

c Comparison states further excluded Pennsylvania and New Hampshire from the Northeast Region of the U.S. Census Division.

d Comparison states included all the 27 states in the U.S. without RCLs at the end of the sample period, which ended in December 2023.

e Comparison states further excluded neighboring states of Virginia: West Virginia, Kentucky, Tennessee, and North Carolina. BRFSS, Behavioral Risk Factor Surveillance System; RCL, recreational cannabis legalization law.

Analyses stratified by population subgroups showed that relative to comparison states, after passing its RCL, cigarette smoking prevalence increased in Virginia among males (1.75 pp; 95% CI=0.03, 3.47; *p*=0.046 or 10.22% relative increase), persons identified as Hispanic race/ethnicity (6.44 pp; 95% CI=2.78, 10.10; *p* = 0.001 or 64.34% relative increase), persons with high school or lower level of education (3.68 pp; 95% CI=1.29, 6.06; *p*=0.003 or 15.93% relative increase), and individuals aged 21–49 years (2.56 pp; 95% CI=0.60, 4.51; *p*=0.01 or 14.71% relative increase) (Table 3). The authors also observed sizable but statistically insignificant increases in cigarette smoking prevalence among females, African Americans, persons of other race/ethnicity, middle-aged adults (aged 50–64 years), and older people (aged ≥65 years). On the other hand, RCL in Virginia led to a decrease in smoking prevalence among young adults aged 18–20 years (−4.63 pp; 95% CI= −7.96, −1.29; *p*=0.007).

**Table 3:** Estimates of the Association Between Recreational Cannabis Legalization Law and Current Cigarette Smoking in Virginia Among Adults Aged ≥18 Years From 2015 to 2023 by Sociodemographic Groups

Population subgroup	Difference-in-differences estimate (95% CI), percentage points^a^	Current cigarette smoking prevalence in Virginia before passing RCL, %	Relative changes, %^b^	*p*-value	BRFSS unweighted sample size, *n*
Sex					
Males	1.75 (0.03, 3.47)	17.13	10.22	0.046	429,936
Females	0.99 (−0.30, 2.28)	13.20	7.50	0.1	562,679
Race/ethnicity^c^					
African American	2.69 (−3.02, 8.40)	17.24	15.61	0.4	138,127
Hispanic	6.44 (2.78, 10.10)	10.01	64.34	0.001	66,705
White	0.00 (−0.95, 0.96)	15.71	0.00	1	719,361
Other^d^	2.02 (−3.22, 8.26)	11.34	17.81	0.5	49,060
Educational level^c^					
High school or lower	3.68 (1.29, 6.06)	23.09	15.93	0.003	365,964
Some college	−0.12 (−1.96, 1.71)	15.74	−0.76	0.9	270,296
College or higher	0.15 (−1.41, 1.72)	6.05	2.48	0.8	352,994
Age groups, years					
18–20	−4.63 (−7.96, −1.29)	7.52	−61.55	0.007	21,858
21–49	2.56 (0.60, 4.51)	17.38	14.71	0.01	315,367
50–64	0.63 (−1.75, 3.01)	17.13	3.68	0.6	283,942
≥65	1.29 (−1.12, 3.70)	8.97	14.39	0.3	371,448

a The difference-in-differences estimates show the association between current cigarette smoking and RCL in Virginia using 15 states from the Northeast and Southern U.S. Census regions that did not pass RCLs before the end of the sample period in December 2023 as comparison states. Comparison states were Alabama, Arkansas, Florida, Georgia, Kentucky, Louisiana, Mississippi, New Hampshire, North Carolina, Oklahoma, Pennsylvania, South Carolina, Tennessee, Texas, and West Virginia.

b The relative changes in the current smoking prevalence were calculated by dividing the difference-in-differences estimates in the second column by the current smoking prevalence rates in Virginia in the third column and multiplying by 100.

c There were 21,237 individuals for whom there was no information on their race/ethnicity and 3,367 respondents with no information on education.

d Other included the following races/ethnicities identified in the BRFSS survey: American Indian or Alaska Native, Asian, Pacific Islander, and other race/ethnicity not listed. BRFSS, Behavioral Risk Factor Surveillance System; RCL, recreational cannabis legalization law.

## DISCUSSION

In April 2021, Virginia passed an RCL to allow cannabis possession, sharing, and cultivation of limited quantities of cannabis among adults (aged ≥21 years) without permitting retail dispensary sales. On the basis of BRFSS survey data from 2015 to 2023, the authors found that the RCL in Virginia without retail dispensary sales was associated with a relative increase in cigarette smoking prevalence of 9.5% among adults (aged ≥18 years). Stratified analysis by sociodemographic population subgroups showed increases in cigarette smoking prevalence among males, persons identifying as Hispanic race/ethnicity, persons with high school or lower education, and individuals aged 21–49 years.

The results are generally consistent with those of a previous study finding increases in cigarette consumption after the passage of RCLs^24^ but are contrary to previous study findings of decreased tobacco use^20^^,^^21^ or no association of RCLs with adult cigarette smoking.^8^^,^^22^^,^^23^ The anecdotal evidence of the dramatic increase in cannabis consumption in Virginia after RCL^10^, ^11^, ^12^ suggests that the increase in smoking prevalence the authors observed among the adult population in Virginia after passing its RCL may be due to recreational cannabis and cigarettes being complements. However, further research can investigate the co-use of cannabis and cigarettes after the passage of an RCL in Virginia.

The findings are also contrary to conclusions in previous studies that have suggested that RCLs impact cannabis use largely through retail dispensary sales and that spillover effects on the use of other substances may not be visible until retail sales commence.^8^^,^^22^ Inconsistencies in the results of studies on the effects of RCLs on smoking prevalence could be addressed in future research that accounts for several factors. These factors could include the timing of RCLs being passed versus implemented, the start of retail dispensary sales, other sources of heterogeneity in legislation (e.g., penalties for violations, level of enforcement), individual-level patterns of cannabis use (experimental versus habitual) before and after passage, and sources for obtaining cannabis. Heterogeneity in the passage and implementation of legislation across states suggests that state-centric analyses with appropriate comparison states may help identify which aspects of RCLs are most relevant to changes in cigarette smoking prevalence in these states.

Little is known about the pathways through which RCLs without the component of retail dispensary sale may influence the use of other substances such as cigarettes. RCLs reduce the stigma associated with using cannabis and perceptions of its health risks.^9^ Even in the absence of retail dispensary sales, the increased acceptability of cannabis associated with the passage of an RCL may encourage experimental or more regular use of cannabis sourced from one’s own or others’ cultivation of cannabis plants. This in turn could drive increases in smoking propensity for some individuals to co-use with cannabis (if cannabis and cigarettes are complements), whereas others might switch to cigarettes (if cannabis and cigarettes are substitutes). Future investigations that follow individual smoking behavior over time can compare between the RCL-associated smoking outcomes in Virginia and states that passed RCLs with concurrent retail dispensary sales and help determine whether allowance of retail sales without lag is advisable or not in minimizing the unintended adverse effects on cigarette smoking behavior. This information would be valuable for states that may be considering an RCL.

The authors also observed that among young adults aged 18–20 years, the law was associated with a decrease in smoking prevalence. This decrease is expected for individuals from this age group because they were ineligible to possess cannabis under the RCL and not permitted to purchase cigarettes under the Tobacco 21 Law.^38^ Indeed, studies have shown that different individuals may be using cannabis for various purposes and that cannabis may be a complement or substitute for tobacco depending on age^13^ or sociodemographic characteristics.^22^ This finding also supports the notion that synchronized tobacco and cannabis regulatory efforts can help protect public health.

### Limitations

Some limitations of the study include BRFSS survey participants’ potential recall bias, and the authors’ inability to follow individual smoking behavior over time from cross-sectional data and account for unmeasured person-level heterogeneity. Future research can address this using more detailed longitudinal data from Virginia and comparison states. Furthermore, the COVID-19 pandemic may confound the effect of RCL on cigarette smoking. However, the sensitivity analyses suggest that the pandemic did not affect the robustness of the results.

## CONCLUSIONS

The adoption of an RCL without allowance of retail dispensary sales of cannabis, similar to that of Virginia, may induce increased cigarette smoking overall and among some sociodemographic population subgroups (e.g., persons identifying as Hispanic race/ethnicity, persons with high school or lower education, and individuals aged 21–49 years). States considering the adoption of an RCL with no authorized legal cannabis sales may need to strengthen tobacco control efforts for populations at risk of increased smoking prevalence.
